# Arterial and venous phase evaluation in fast kV-switching dual-energy CT for detecting acute small bowel ischemia caused by small bowel obstruction

**DOI:** 10.1007/s11604-025-01873-8

**Published:** 2025-09-13

**Authors:** Masazumi Matsuda, Motoko Konno, Takahiro Otani, Tomoki Tozawa, Kento Hatakeyama, Toshiki Murata, Emika Murasawa, Daichi Sugawara, Junichi Arita, Hajime Nakae, Naoko Mori

**Affiliations:** 1 Department of Radiology, Akita University Graduate School of Medicine, 1-1-1 Hondo, Akita, 010-8543 Japan; 2 Department of Gastroenterological Surgery, Akita University Graduate School of Medicine, 1-1-1 Hondo, Akita, 010-8543 Japan; 3 Department of Emergency and Critical Care Medicine, Akita University Graduate School of Medicine, 1-1-1 Hondo, Akita, Japan

**Keywords:** Dual-energy CT, Acute small bowel ischemia, Small bowel obstruction, Arterial phase

## Abstract

**Purpose:**

To compare the diagnostic performance of fast kV-switching dual-energy CT (DECT) parameters, including virtual monochromatic imaging (VMI) and iodine mapping, in the arterial and venous phases for detecting surgically confirmed acute small bowel ischemia (ASBI) in cases suspected as ASBI on conventional visual CT findings.

**Materials and methods:**

Thirty-two patients with conventional visual CT findings suggesting possible or suspected ASBI caused by small bowel obstruction (SBO) were included. Two radiologists independently placed ten cursors on visually hypo-perfused bowel wall regions to measure CT values at 70 keV, 40 keV, and iodine quantity. Patients were categorized into surgically confirmed ASBI (*n* = 12) and non-confirmed ASBI (*n* = 20). ROC analysis assessed diagnostic performance, and inter-observer reliability was evaluated using intra-class correlation coefficients (ICC).

**Results:**

For both observers, the CT value at 40 keV and iodine quantity in the arterial phase, as well as iodine quantity in the venous phase, was significantly different between surgically confirmed ASBI and non-confirmed ASBI groups (*p* < 0.05). Iodine quantity consistently yielded the highest AUC among the evaluated parameters in each phase although the differences compared to 70-keV VMI were not statistically significant. The parameters in the arterial phase tended to demonstrate higher AUCs than those in the venous phase. Inter-observer agreement was moderate to substantial (ICC 0.585–0.741), while intra-observer agreement was substantial to almost perfect (ICC 0.733–0.940).

**Conclusions:**

DECT parameters, such as the CT value at 40-keV and the iodine quantity, were effective in differentiating surgically confirmed ASBI from non-confirmed ASBI in SBO cases with suspected ischemia. Iodine quantity showed the highest diagnostic performance among all evaluated parameters. Although the differences were not statistically significant, arterial phase parameters generally yielded higher AUCs than those in the venous phase, suggesting the potential utility of arterial phase DECT in the detection of ASBI.

## Introduction

Acute small bowel ischemia (ASBI) is a complex condition caused by the interruption of blood flow to the small bowel, which can result from mesenteric artery occlusion, mesenteric vein thrombosis, non-occlusive mesenteric ischemia, and strangulated small bowel obstruction (SBO) [1]. The mortality rate of ASBI is high, exceeding 50% [2–5]. Prompt intervention is critical to avoid poor outcomes in cases of ASBI; however, clinical signs and laboratory findings alone are insufficient to reliably predict ASBI, making CT imaging an essential diagnostic tool [6, 7]. In this study, the abbreviation SBO refers to small bowel obstruction in general, while the term'strangulated SBO'is used specifically to indicate cases of obstruction accompanied by compromised blood flow.

In the diagnosis of ASBI, particularly in cases of strangulated SBO, vascular occlusion may not be visualized on CT. Instead, evaluation relies on assessing the bowel wall and associated findings. Conventional visual CT findings of ASBI include reduced bowel enhancement, mural thickening, bowel-wall edema, unusual course of the mesenteric vasculature, diffuse mesenteric haziness, ascites, pneumatosis, and increased bowel wall attenuation on non-contrast-enhanced images [4]. The diagnostic performance of the conventional visual CT findings for ASBI was sensitivity ranging from 73 to 100% and specificity from 61 to 100% [8, 9]; however, this reported diagnostic performance might overestimate the actual effectiveness of CT in clinical practice. One study reported a sensitivity as low as 14.8% in prospective evaluation and 51.9% in retrospective evaluation [10].

Dual-energy CT (DECT) has emerged as a promising imaging technology that may provide additional diagnostic information. DECT utilizes the fact that X-ray mass attenuation coefficients vary depending on the substance, using two distinctly different energy levels (e.g., 80 and 140 kVp) to allow discrimination of substances [11]. DECT post-processing methods, such as virtual monochromatic imaging (VMI) and iodine mapping, have already shown utility in liver, kidney, and pancreatic imaging [12–14]. Moreover, the potential of DECT in bowel disease diagnosis is increasingly recognized [15]. A recent study including small and large bowels found that the sensitivity for detecting acute bowel ischemia increased from 63.6% with conventional CT to 100% with 40 keV VMI and 81.8% with iodine mapping [16]. However, this study was limited to the portal venous phase, leaving room for further investigation. The most appropriate scanning phase for detecting acute bowel ischemia remains uncertain. In single-energy CT, the arterial phase has been shown to be superior to the portal venous phase in detecting ASBI [17]. There was no study comparing the diagnostic performance of DECT between arterial and venous phases for detecting ASBI to our knowledge.

Further, studies reporting the usefulness of DECT for detecting acute bowel ischemia show variability in the selection of the study population. Some studies included cases where DECT was performed specifically for the evaluation of acute bowel ischemia [18], while others matched non-ischemic cases with ischemic cases for comparison [19]. Some studies included both small and large bowel ischemia [16, 19], whereas others focused exclusively on small bowel ischemia [20]. In our clinical practice, small bowel diseases and large bowel diseases are managed separately. Further, in small bowel disease, ASBI caused by SBO, or strangulated SBO are managed separately from vascular disease. For small bowel diseases, a two-step evaluation approach is employed. In the first step, conventional visual CT findings are used to identify cases where ASBI is suspected. In the second step, VMI and iodine mapping are generated using DECT. Therefore, it is important to assess the diagnostic performance of DECT for detecting ASBI in SBO cases where ASBI is suspected by conventional visual CT findings.

The purpose of our study was to compare the diagnostic performance of VMI and iodine mapping of DECT in both arterial and venous phases for detecting ASBI in SBO cases with suspected ischemia based on conventional CT findings.

## Materials and methods

### Patients

**Fig. 1:**
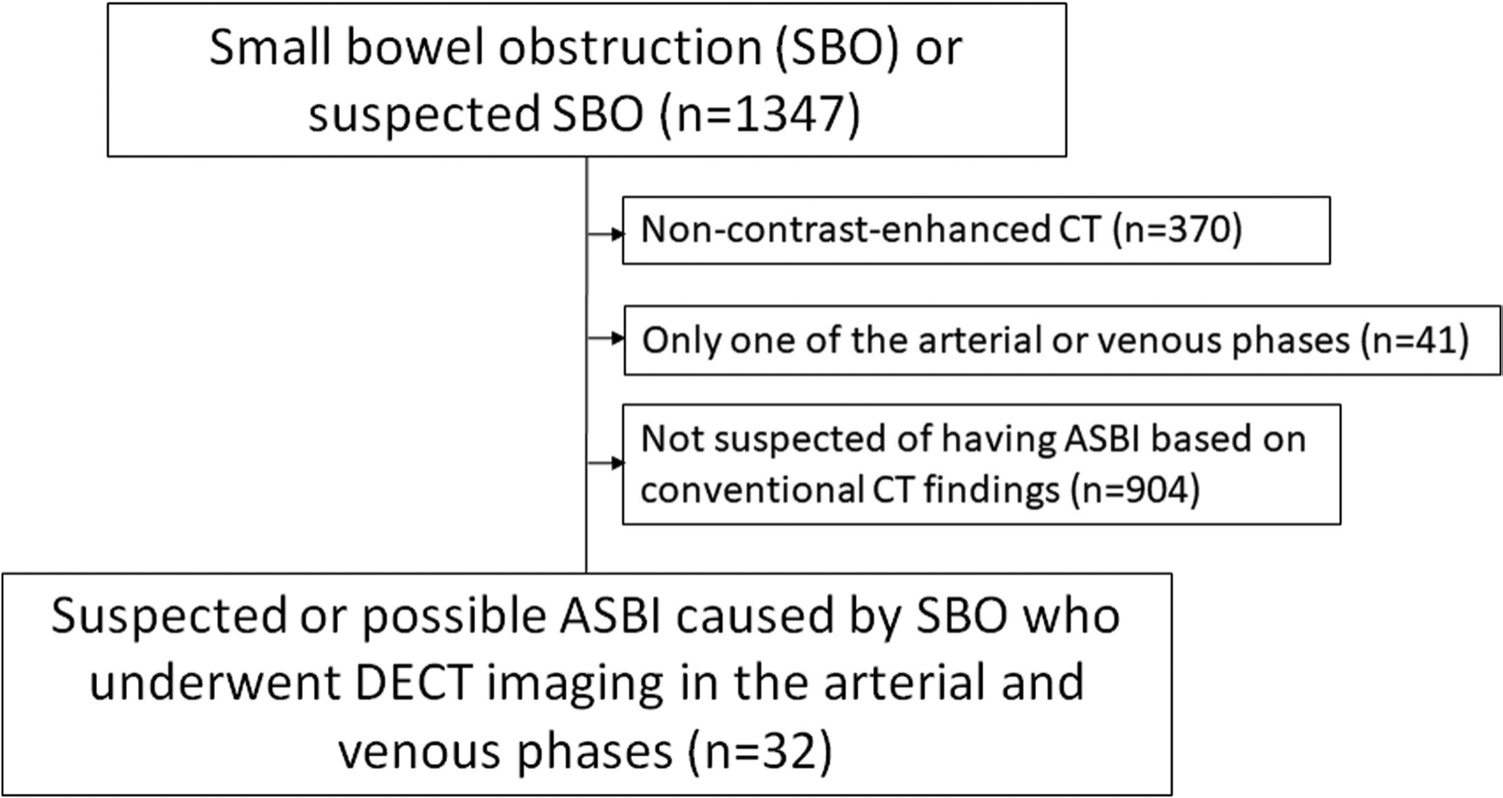
Flowchart illustrating the selection process for the study population. Among 1347 patients with small bowel obstruction (SBO) or suspected SBO, cases were excluded according to the following criteria: those with non-contrast-enhanced CT only (*n* = 370), those with imaging limited to a single phase (arterial or venous; *n* = 41), and those where conventional visual CT findings did not suggest acute small bowel ischemia (ASBI) (*n* = 904). Following the application of these criteria, 32 patients with SBO in whom ASBI was suspected or considered possible based on conventional visual CT findings and who underwent dual-energy CT (DECT) in both the arterial and venous phases were included in the final analysis

This retrospective study was approved by the institutional review board, with a waiver of the requirement for informed consent. The inclusion criteria were patients who underwent DECT including arterial and venous phases with suspected SBO and were deemed to have a possible or suspected ASBI. To identify eligible cases, we conducted a search of our radiology reporting system and medical records from January 2018 to February 2024 for reports containing the terms “ SBO “ or “suspected SBO “ (*n* = 1347). Among these, exclusion criteria were applied as follows: First, cases were excluded if imaging consisted only of non-contrast-enhanced CT (*n* = 370). Second, cases were excluded if only one phase, either arterial or venous, was available (*n* = 41). Third, we excluded cases where conventional visual CT findings did not suggest ASBI (*n* = 904). Finally, 32 patients with SBO in whom ASBI was suspected or considered possible based on conventional visual CT findings and who underwent DECT in both the arterial and venous phases were included in the final analysis (Fig. 1). The radiology reporting system was used to extract which conventional visual CT findings raised suspicion for ASBI, including reduced bowel enhancement, mural thickening, bowel-wall edema, unusual course of the mesenteric vasculature, diffuse mesenteric haziness, ascites, pneumatosis, and increased bowel wall attenuation on non-contrast-enhanced images. These findings were reviewed by two radiologists with 20 and 8 years of experience in abdominal imaging (N.M. and M.M., respectively), who also reviewed the corresponding CT images together. The presence or absence of each finding was confirmed by consensus for all included cases. There was no discrepancy between these interpretations and the original radiology reports. However, for cases not suspected of having ASBI based on conventional CT findings (*n* = 904), no such review was performed. Patient characteristics, including age, gender, surgery for the present disease, height, body weight, and white blood cell count, were obtained from the hospital information system. We categorized cases based on medical records into two groups: “surgically confirmed ASBI,” defined as cases with surgically confirmed small bowel necrosis, and “non-confirmed ASBI,” defined as cases that either improved with observation or had no small bowel necrosis upon surgical evaluation, including cases where strangulation release was performed. In this study, surgically confirmed small bowel necrosis was defined as ischemia (ASBI), and although some cases in the “non-confirmed ASBI” group may have had ischemia at the time of CT imaging, they were classified as “non-confirmed ASBI”.

### Image acquisition

All patients were scanned using a Revolution CT scanner (GE Healthcare Japan, Tokyo, Japan), a fast kV-switching Dual-Energy CT system. The imaging protocol consisted of non-contrast-enhanced, arterial phase, and venous phase acquisitions without the use of oral contrast material. DECT data were acquired with fast kV-switching (80/140 kV) using Gemstone Spectral Imaging (GSI) mode, using a detector configuration of 128 × 0.625 mm and a Noise Index of 9.7. The reference tube current was set at 202.5 mAs (405 mA × 0.5 s), and the gantry rotation time was 0.5 s. The helical pitch was fixed at 0.922, and the field of view (FOV) was 34.5 cm. Images were reconstructed at two different time points for each acquisition phase. For images obtained 40 s after contrast injection (arterial phase), the reconstructed section thickness was 2.5 mm and 1.25 mm, using deep learning image reconstruction (DLIR) at low and medium strength levels. For images acquired 100 s after contrast injection (venous phase), the reconstructed section thickness was 5.0 mm and 1.25 mm, also utilizing DLIR at low and medium strength levels. Nonionic, low-osmolality, iodine-based contrast agents were used: Iopamidol (Iopamidol®, Fuji Pharma Co., Ltd., 300–370 mgI/mL), Iomeprol (Iomeron®, Bracco Imaging, 300–350 mgI/mL), Iopromide (Iopromide®, Bayer AG, 300–370 mgI/mL), and Ioversol (Optiray®, Guerbet, 320–350 mgI/mL). Specific agent and concentration were selected according to availability and patient condition, and the iodine dose was adjusted to approximately 500–600 mgI/kg body weight. The injection rates ranged from 1.6 to 3.1 mL/s, with a total contrast medium volume of 75–120 mL adjusted based on the patient body weight. Detailed iodine dose, injection rate of contrast material, volume of contrast material, and injection time were obtained from the radiology information system.

### Image analysis

**Fig. 2:**
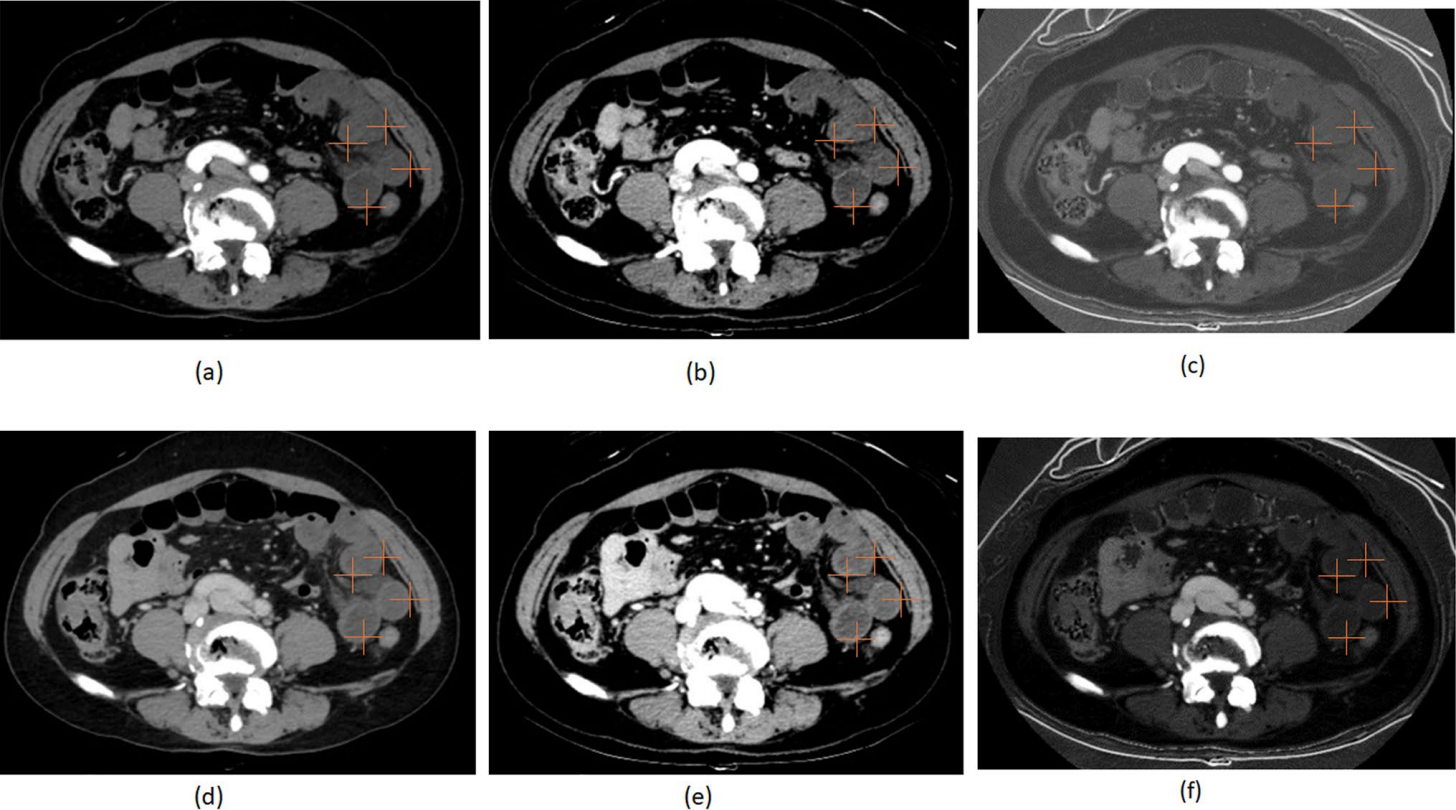
Representative images from a 54-year-old woman with surgically confirmed acute small bowel ischemia (ASBI) who required resection of 50 cm of small bowel due to necrosis. The two radiologists independently placed ten cursors on the small bowel wall in areas appearing to have reduced contrast enhancement in the arterial phase (**a–c** 70-keV, 40-keV, iodine quantity) and venous phase (**d–f** 70-keV, 40-keV, iodine quantity), ensuring that the cursors were placed at the same anatomical locations on the 70-keV images, 40-keV images, and iodine maps within each phase. In this slice, four cursors are shown. The mean CT values in the arterial phase were 27.0 HU at 70-keV and 51.3 HU at 40-keV, with a mean iodine quantity of 4.0 μg/cm^3^. In the venous phase, the mean CT values were 35.7 HU at 70-keV and 53.4 HU at 40-keV, with a mean iodine quantity of 4.0 μg/cm^3^

**Fig. 3:**
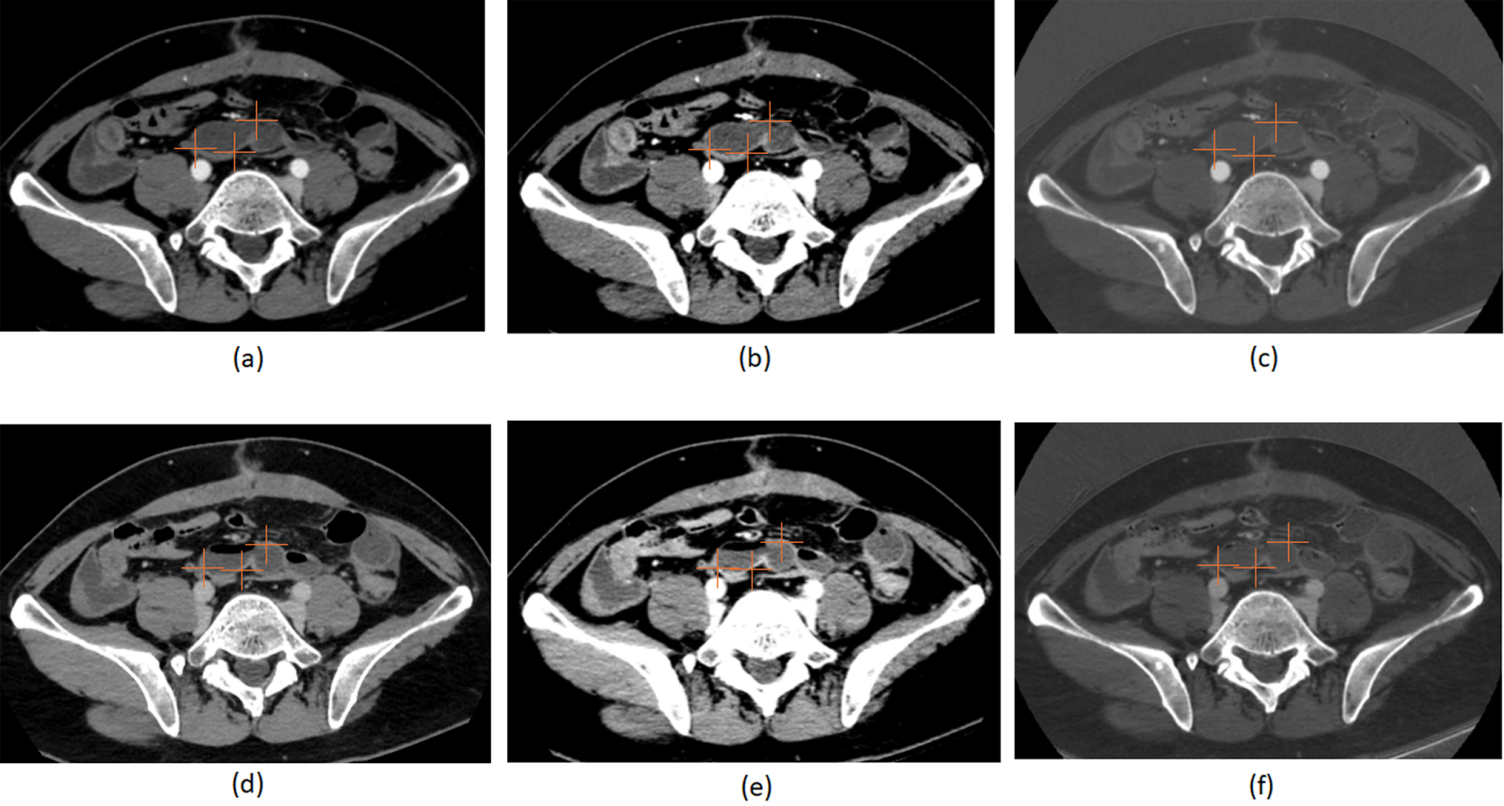
Representative images from a 40-year-old man in the non-confirmed acute small bowel ischemia (ASBI) group who improved with conservative management (fasting). Ten cursors were placed on the small bowel wall in areas appearing to have reduced contrast enhancement in the arterial phase (**a–c** 70-keV, 40-keV, iodine quantity) and venous phase (**d–f** 70-keV, 40-keV, iodine quantity). In this slice, three cursors are shown as examples. In the arterial phase, the mean CT values were 48.0 HU at 70-keV and 105.2 HU at 40-keV, with a mean iodine quantity of 9.9 μg/cm^3^. In the venous phase, the mean CT values were 47.8 HU at 70-keV and 84.6 HU at 40-keV, with a mean iodine quantity of 12.0 μg/cm^3^

DECT data were post-processed using dedicated software (AW Server 3.2 Ext.2.0, GE Healthcare Japan, Tokyo, Japan). The 70-keV VMI, 40-keV VMI, and iodine-map images were generated for both the arterial and venous phases. Iodine maps were automatically generated using the Gemstone Spectral Imaging (GSI) software integrated into the CT scanner (GE Healthcare). The GSI technique employs a fast kV-switching dual-energy acquisition method (80/140 kVp), allowing for material decomposition based on the energy-dependent attenuation properties of iodine. Iodine quantity (μg/cm^3^) was calculated using a two-material decomposition algorithm, which assumes water and iodine as basis materials. The algorithm estimates the iodine content for each voxel by analyzing the attenuation differences between low- and high-energy data. The resulting iodine maps were post-processed and visualized using the AW Server (GE Healthcare). The maps were then exported as monochromatic DICOM images and transferred to a Synapse Picture Archiving and Communication System (PACS) viewer (Fujifilm Medical Co., Ltd., Tokyo, Japan). The 70-keV VMI in DECT was made as a comparable alternative to conventional 120-kVp CT [21, 22]. These images were reconstructed with a slice thickness of 1.25 mm. The CT images were retrospectively reviewed by two radiologists with 20 and 8 years of experience (* and **, respectively) in abdominal imaging. For each phase of each case, the two radiologists independently placed ten cursors on voxels of the small bowel wall that appeared to have reduced contrast enhancement (Fig. 2 and 3), ensuring that the cursors were placed at the same anatomical locations on the 70-keV images, 40-keV images, and iodine maps within each phase. In cases where reduced bowel enhancement was not clearly observed, the cursor was placed on the segment of the bowel wall that appeared to have the relatively lowest enhancement, based on visual assessment. Cursor placement was performed in the same manner for both the arterial and venous phases. However, manual adjustments were made in cases of bowel wall motion. In cases where the bowel wall was thickened, the cursors were positioned on the mucosal side. When the bowel wall was not thickened, the wall was indeed very thin; however, the cursor was still placed as close to the mucosal side as possible. The CT values or iodine quantities measured at these ten locations were averaged for analysis. To assess intra-observer reliability, the two observers performed a second round of cursor placements after a one-month interval. Inter-observer reliability between the two readers was assessed using the intra-class correlation coefficient (ICC). Intra-observer reliability for each reader was also evaluated using the ICC. An r value of 1.0 was deemed to indicate perfect agreement; 0.81–0.99, almost perfect agreement; 0.61–0.80, substantial agreement; 0.41–0.60, moderate agreement; 0.21–0.40, fair agreement; and 0.20 or less, slight agreement.

### Statistical analysis

Continuous variables, including age, height, body weight, white blood cell count (WBC), injection rate of contrast material, volume of contrast material, injection time, CT values in 70-keV and 40-keV VMI, and iodine quantities at arterial and venous phases, were compared between surgically confirmed ASBI and non-confirmed ASBI groups using the Mann–Whitney U test. In addition, iodine dose per kilogram of body weight (mgI/kg) was calculated for each patient and compared between the surgically confirmed ASBI group and the non-confirmed group using the Mann–Whitney U test. Categorical variables, such as gender, surgery, and the presence or absence of conventional visual CT findings, were compared between the two groups using the chi-square test. Receiver operating characteristic (ROC) curve analysis was performed to assess the diagnostic performance of CT values in 70-keV and 40-keV VMI, as well as iodine quantities, at the arterial and venous phases. The area under the curve (AUC) of the ROC curve for CT values in 40-keV VMI and iodine quantities were compared to that for CT values in 70-keV VMI at the arterial and venous phases, as 70-keV VMI in DECT is recognized as a comparable alternative to conventional 120-kVp CT [21, 22]. In this process, we assessed whether DECT demonstrated significant advantages over conventional CT in detecting ASBI. Additionally, the highest AUC observed in the arterial phase was compared to the highest AUC observed in the venous phase. All statistical analyses were conducted using JMP Pro 17 (SAS Institute Inc., Cary, NC, USA). A *p* value < 0.05 was considered statistically significant.

## Results

No significant differences were observed in age, gender, height, body weight, or CT contrast material injection parameters; however, the surgery rate was significantly higher in the surgically confirmed ASBI group than in the non-confirmed ASBI group (*p* = 0.002), and the white blood cell count was significantly elevated in the surgically confirmed ASBI group compared to the non-confirmed ASBI group (*p* = 0.010) (Table 1). In 12 of the 20 cases in the non-confirmed ASBI group, surgery was performed; however, bowel resection was not required. Instead, surgical release of adhesions was performed, resulting in improvement of SBO-related symptoms. Among the 12 cases that underwent surgery and the 8 that did not, there was no significant difference in the frequency of reduced bowel enhancement (6/12 vs. 4/8, *p* = 1.0). On conventional visual CT findings, reduced bowel enhancement was observed in 100% of the surgically confirmed ASBI group, a significantly higher rate than the 50% observed in the non-confirmed ASBI group (*p* = 0.003) (Table 1).

**Table 1:** Comparison of background factors, CT contrast injection parameters, and conventional visual CT findings between surgically confirmed ASBI and non-confirmed ASBI

Parameter	Surgically confirmed ASBI (*n* = 12)	Non-confirmed ASBI (*n* = 20)	*p* value
Age	65.3 ± 16.2	61.9 ± 19.3	0.696
Gender (Male/Female) (number (%))	8 (66.7)/4 (33.3)	14 (70.0)/6 (30.0)	1.000
Surgery (number (%))	12 (100.0)	12 (60.0)	0.002*
Height (cm)	160.5 ± 5.4	163.3 ± 8.5	0.300
Body weight (kg)	53.6 ± 12.2	50.0 ± 9.9	0.417
White blood cell count	11,542 ± 3121	8110 ± 3183	0.010*
CT contrast material injection			
Iodine dose (mgI/kg)	527 ± 88	528 ± 111	0.923
Injection rate of contrast material (ml/sec)	2.71 ± 0.59	2.67 ± 0.63	0.859
Volume of contrast material (ml)	84.8 ± 22.3	81.6 ± 16.00	0.754
Injection time (sec)	31.1 ± 2.9	31.6 ± 7.1	0.661
Conventional visual CT findings (absence/presence) (number (%))			
Reduced bowel enhancement	0 (0.0)/12 (100.0)	10 (50.0)/10 (50.0)	0.003*
Mural thickening	10 (83.3)/2 (16.7)	18 (90.0)/2 (10.0)	0.610
Unusual course of the mesenteric vasculature	8 (66.7)/4 (33.3)	16 (80.0)/4 (20.0)	0.421
Diffuse mesenteric haziness	1 (8.3)/11 (91.7)	7 (35.0)/13 (65.0)	0.102
Ascites	1 (8.3)/11 (91.7)	0 (0.0)/20 (100.0)	0.220
Pneumatosis	12 (100.0)/0 (0.0)	18 (90.0)/2 (10.0)	0.285
Bowel-wall oedema	7 (58.3)/5 (41.7)	14 (70.0)/6 (30.0)	0.523
Increased bowel wall attenuation on non-contrast-enhanced images	7 (58.3)/5 (41.7)	17 (85.0)/3 (15.0)	0.102

*ASBI* Acute small bowel ischemia

*Statistically significant (*p* 0.05)

**Fig. 4:**
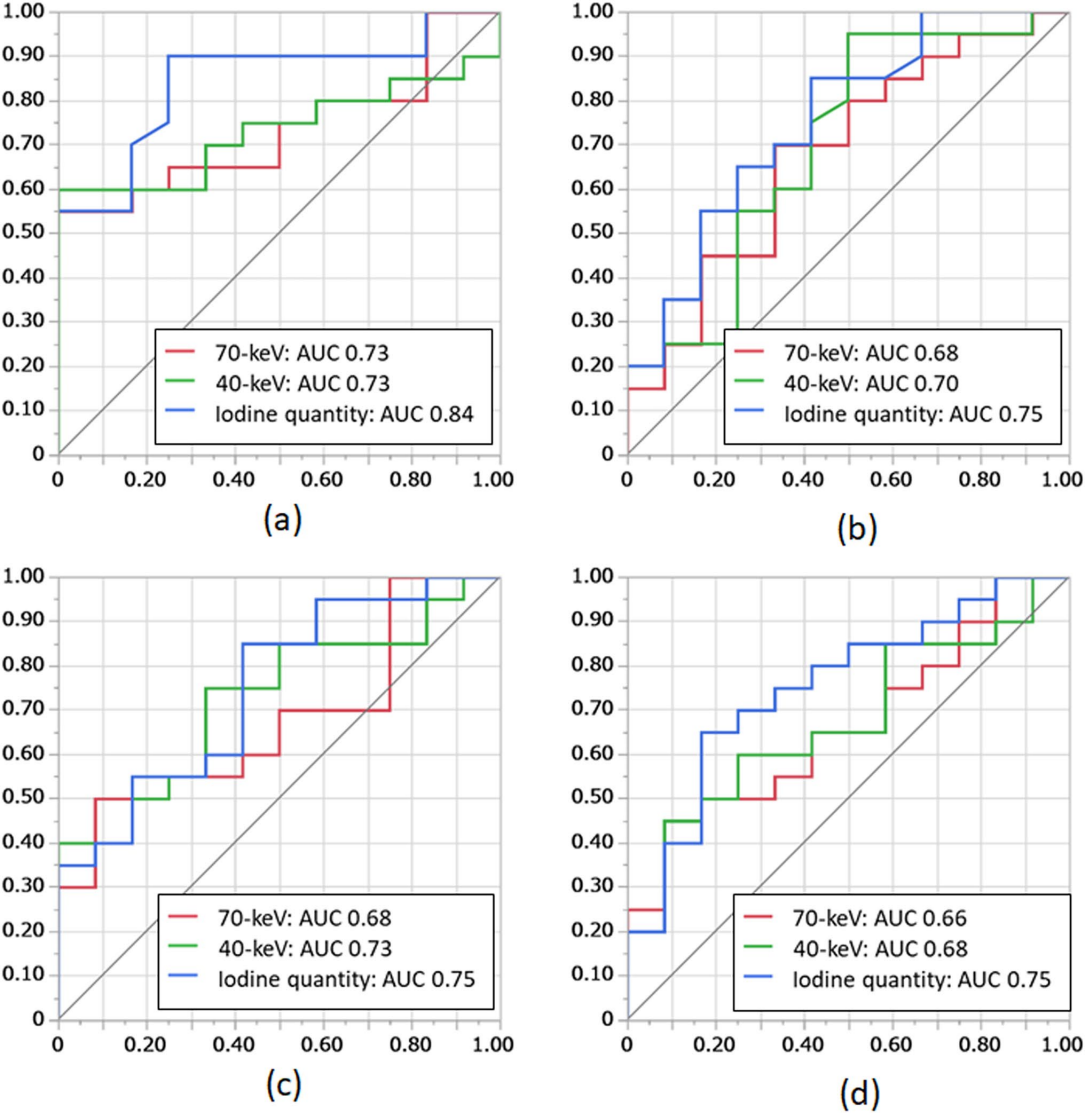
Receiver operating characteristic (ROC) curves of dual-energy CT (DECT) parameters for distinguishing surgically confirmed acute small bowel ischemia (ASBI), shown separately for each of the two observers. **a** Observer 1—Arterial phase: The area under the curves (AUCs) of iodine quantity (0.84) was highest. **b** Observer 1—Venous phase: The AUC of iodine quantity (0.75) was highest. **c** Observer 2—Arterial phase: The AUC of iodine quantity (0.75) was highest. **d** Observer 2—Venous phase: The AUC of iodine quantity (0.75) was highest

**Fig. 5:**
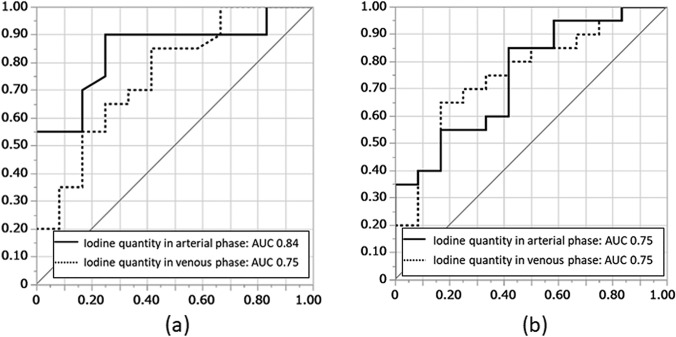
Receiver operating characteristic (ROC) curves comparing the highest area under the curve (AUC) parameters in the arterial and venous phases for distinguishing surgically confirmed acute small bowel ischemia (ASBI). **a** Observer 1-The curves represent iodine quantity in the arterial phase and iodine quantity in the venous phase. There was no significant difference between the AUCs (*p* = 0.195). **b** Observer 2- The curves represent iodine quantity in the arterial phase and iodine quantity in the venous phase. There was no significant difference between the AUCs (*p* = 0.945)

For both observers, the CT value of 40-keV and iodine quantity in the arterial phase, as well as iodine quantity in the venous phase, were significantly different between surgically confirmed ASBI and non-confirmed ASBI groups (Table 2). For both observers, the iodine quantity in the arterial and venous phases showed the highest AUC among the evaluated parameters in each phase. However, the difference was not statistically significant when compared to the CT value of 70-keV (Table 2, Fig. 4). While there was no significant difference in AUC between the arterial and venous phases, the parameters in the arterial phase tended to show higher AUCs than those in the venous phase (Table 2, Fig. 5).

**Table 2:** Comparison of DECT parameters at arterial and venous phases between surgically confirmed ASIB and non-confirmed ASBI

Parameter	Surgically confirmed ASBI (*n* = 12)	Non-confirmed ASBI (*n* = 20)	*p* value	AUC	Comparison to 70-keV (*p* value)
Observer 1					
Arterial phase					
CT values in 70-keV (HU)	32.2 ± 10.8	44.1 ± 14.8	0.031*	0.73	-
CT values in 40-keV (HU)	69.7 ± 20.2	100.0 ± 43.6	0.031*	0.73	1.000
Iodine quantity (μg/cm^3^)	4.5 ± 2.2	9.8 ± 5.6	0.001*	0.84	0.139
Venous phase					
CT values in 70-keV (HU)	38.4 ± 13.9	47.4 ± 11.8	0.098	0.68	-
CT values in 40-keV (HU)	79.9 ± 34.3	105.9 ± 35.7	0.067	0.70	0.804
Iodine quantity (μg/cm^3^)	6.83 ± 3.43	10.9 ± 4.8	0.020*	0.75	0.399
Observer 2					
Arterial phase					
CT values in 70-keV (HU)	30.5 ± 11.4	40.4 ± 13.4	0.100	0.68	-
CT values in 40-keV (HU)	60.4 ± 22.1	80.9 ± 28.7	0.037*	0.73	0.469
Iodine quantity (μg/cm^3^)	5.1 ± 1.8	7.7 ± 3.1	0.021*	0.75	0.429
Venous phase					
CT values in 70-keV (HU)	38.6 ± 12.7	47.9 ± 15.0	0.129	0.66	-
CT values in 40-keV (HU)	75.2 ± 27.2	96.7 ± 35.7	0.106	0.68	0.903
Iodine quantity (μg/cm^3^)	6.9 ± 3.0	10.3 ± 3.9	0.019*	0.75	0.411

*DECT* Dual-energy CT, *ASBI* Acute small bowel ischemia, *AUC* Area under the curve

*Statistically significant (*p* 0.05)

Regarding inter-observer reliability, the ICC values for arterial phase parameters showed substantial agreement, while venous phase parameters, ICC values demonstrated moderate to substantial agreement. For intra-observer reliability, both observers showed substantial to almost perfect agreement (Table 3).

**Table 3:** Inter- and intra-observer reliability in each parameter at arterial and venous phases

Parameter	Inter-observer reliability	Intra-observer reliability (observer 1)	Intra-observer reliability (observer 2)
Arterial phase			
CT values in 70-keV (HU)	0.637 [0.375, 0.804]	0.879 [0.768, 0.939]	0.896 [0.799, 0.948]
CT values in 40-keV (HU)	0.741 [0.533, 0.865]	0.873 [0.756, 0.936]	0.920 [0.843, 0.960]
Iodine quantity (μg/cm^3^)	0.655 [0.401, 0.815]	0.940 [0.882,0.970]	0.912 [0.829, 0.956]
Venous phase			
CT values in 70-keV (HU)	0.585 [0.302, 0.773]	0.733 [0.520, 0.860]	0.851 [0.717, 0.924]
CT values in 40-keV (HU)	0.613 [0.341, 0.791]	0.880 [0.768, 0.939]	0.822 [0.666, 0.909]
Iodine quantity (μg/cm^3^)	0.592 [0.311, 0.777]	0.804 [0.636, 0.899]	0.753 [0.552, 0.871]

## Discussion

Previous studies have demonstrated that 70-keV VMI of DECT provides CT values equivalent to those of conventional 120-kVp images [21, 22]. Therefore, 70-keV VMI has been recognized as a comparable alternative to conventional 120-kVp CT. Darras et al. reported that 70-keV VMI optimizes contrast-to-noise ratio for small bowel mural enhancement and improves observer confidence; however, it does not improve diagnostic accuracy for ASBI compared to conventional 120-kVp imaging [20]. In the present study, compared to the period of Darras's report, the introduction of noise reduction technologies, such as deep learning-based image reconstruction (DLIR), may have improved image quality by reducing noise in 40-keV VMI [23]. DLIR has demonstrated superior noise reduction capabilities while maintaining image quality, particularly at low keV settings [24, 25]. This improvement may explain why, as observed in this study, the diagnostic performance of 40-keV VMI tends to be superior to that of 70-keV VMI in detecting surgically confirmed ASBI during the arterial and venous phases.

In our study, pneumatosis was observed in two cases within the non-confirmed ASBI group but in none of the surgically confirmed ASBI cases. Although this distribution appears paradoxical in relation to disease severity, the difference was not statistically significant (*p* = 0.285). The reduced bowel enhancement was observed in 100% of the surgically confirmed ASBI group, a significantly higher rate than the 50% observed in the non-confirmed ASBI group (*p* = 0.003). In this study, the radiology reporting system was used to extract conventional visual CT findings that raised suspicion for ASBI, including reduced bowel enhancement. These findings were reviewed by two radiologists and no discrepancy was found between these interpretations and the original radiology reports. However, for the patients outside the included cohort (*n* = 904), who were not suspected of having ASBI based on conventional CT findings, the conventional visual CT findings were not reviewed. This represents a limitation of the present study.

Our study demonstrated that the arterial phase tends to provide superior diagnostic performance compared to the venous phase for detecting surgically confirmed ASBI, regardless of the observer, although the difference was not statistically significant. The arterial phase captures early perfusion abnormalities caused by compromised arterial flow, which are key indicators of ischemia [26–28]. In contrast, the venous phase often reflects secondary Changes, such as venous congestion and bowel wall edema, which are less specific for identifying ischemic damage. The findings from observer 1, which demonstrated that the diagnostic performance of iodine quantity in the arterial phase was highest for detecting surgically confirmed ASBI, are consistent with the findings of Kok et al. [29]. Their research using single-energy CT showed the value of the arterial phase, indicating it improves the detection of ischemic bowel conditions with greater specificity and sensitivity compared to the portal venous phase [29]. This consistency demonstrates the ability of the arterial phase to visualize early perfusion deficits, such as reduced iodine uptake. Additionally, DECT technologies, such as iodine mapping and VMI (e.g., 40 keV), further improve the visibility of ischemic regions in the arterial phase by increasing contrast conspicuity. While the venous phase has traditionally been used in abdominal imaging due to its utility in identifying vascular congestion and delayed enhancement patterns, its limitations in detecting early ischemia might make it less effective in cases of ASBI. Nakamura et al. stated that in patients with SBO, the luminal pressure rises, initially exceeding venous flow and subsequently arterial flow, ultimately leading to bowel ischemia [6]. Thus, it is not surprising that the arterial phase of DECT was useful for assessing whether arterial flow had finally decreased or not.

The fixed delay technique with 40- and 100-s delays used in this study has limitations in accurately capturing the arterial phase. Instead, the bolus-tracking method enables more precise arterial phase timing by individualizing scan timing based on real-time contrast enhancement monitoring. Optimal contrast-enhanced abdominal CT imaging with bolus-tracking involves placing a region of interest (ROI) in the abdominal aorta and initiating monitoring scans after injecting contrast [30]. Individual variations in cardiac function and venous access conditions cannot be accounted for with fixed delay protocols, making bolus-tracking methods more accurate for arterial phase imaging compared to fixed delay techniques [31, 32]. Future studies investigating the use of the bolus-tracking method to evaluate iodine quantity in the arterial phase for predicting surgically confirmed ASBI will be needed.

In our imaging analysis method, the two radiologists independently placed ten cursors on voxels within the small bowel wall that appeared to have reduced contrast enhancement. Given the challenges of placing an ROI with a measurable area on the extremely thin small bowel wall, measurements were performed on ten specific pixels using cursors. The inter-observer reliability for arterial and venous phase parameter was moderate to substantial agreement. However, the relatively low inter-observer agreement remains a challenge. Future research focuses on developing automated tools for measuring CT values and iodine quantification in the small bowel wall will be needed to improve diagnostic accuracy and inter-observer reliability.

The significantly higher surgery rate and white blood cell count in the surgically confirmed ASBI group than in the non-confirmed ASBI group indicate the systemic inflammatory response and severity of ischemia. These findings are consistent with previous reports that showed the importance of combining laboratory and imaging findings for the comprehensive evaluation of ASBI [33, 34]. However, our present study is limited by the lack of additional laboratory data, such as base excess and lactate levels, which are known to be useful markers for predicting ischemia in cases of ASBI [35, 36]. The unavailability of these laboratory data limits the results of our study. In 12 of the 20 cases in the non-confirmed ASBI group, surgery was performed. Bowel resection was not required in these cases. Instead, surgical release of adhesions was performed, and the SBO-related symptoms improved. Postoperative evaluation using DECT was not performed in these cases, and the impact of the surgical release of adhesions on CT findings was not assessed.

With regard to the study population, there is variation in the literature on the evaluation of ASBI. Because ischemia of SBO is rare, Oberparleiter et al. conducted a case–control study comparing 25 patients with surgically proven acute intestinal ischemia with a control group of 25 sex- and age-matched patients who underwent DECT [19]. Their results showed that the addition of DECT to conventional visual CT findings improved diagnostic accuracy and reliability [19]. On the other hand, a notable characteristic of our study is that the cases included were those classified as “possible” or “suspected” ASBI based on conventional visual CT findings. Indeed, DECT parameters in the arterial and venous phases tended to correspond with the presence or absence of reduced bowel enhancement on conventional visual analysis (Appendix 1). This approach reflects current clinical practice, where VMI and iodine quantity maps from DECT are not automatically generated in routine practice and are instead created additionally when radiologists consider them necessary. Therefore, our findings indicate that in cases classified as “possible” or “suspected” ASBI based on conventional visual CT findings, CT value at 40-keV and iodine quantity in the arterial phase of DECT might be effective for detecting surgically confirmed ASBI. A two-step assessment approach using both conventional visual CT findings and DECT-derived arterial phase parameters may be considered minimally required and beneficial in routine practice. Future research should explore the broader application of conventional visual CT findings and DECT in the comprehensive evaluation of ASBI.

Our study has several limitations. First, the sample size was small, which may limit the generalizability of the findings. Second, this was a retrospective study conducted at a single institution, potentially introducing selection bias. Third, while DECT provides unique information, its availability is still limited in many clinical settings, which may restrict the widespread applicability of our findings. Fourth, we did not perform grading of ischemia. Lamant et al. classified cases into ischemia only and necrosis confirmed by small bowel resection, demonstrating the utility of iodine quantity of DECT [18]. In this study, we defined ASBI as necrosis confirmed by surgical removal of the small bowel, and investigated the ability of DECT to distinguish between surgically confirmed ASBI and non-confirmed ASBI. During surgery, the surgeon observed cases where the color of the small bowel was poor during strangulation and returned to normal after the release of strangulation. These cases were classified as non-confirmed ASBI. In such cases, ASBI may have been present at the time of preoperative DECT imaging, and it may be necessary to grade these cases separately from non-confirmed ASBI. Future studies are needed to investigate the application of arterial phase iodine quantity of DECT for ischemia grading in consecutive cases.

In conclusion, DECT parameters, such as the CT value at 40-keV and the iodine quantity, were effective in differentiating surgically confirmed ASBI from non-confirmed ASBI in SBO cases with suspected ischemia. Iodine quantity showed the highest diagnostic performance among all evaluated parameters. Although the differences were not statistically significant, arterial phase parameters generally yielded higher AUCs than those in the venous phase, suggesting the potential utility of arterial phase DECT in the detection of ASBI.

## Appendix 1. Comparison of parameters in the arterial and venous phases between patients with and without reduced bowel enhancement on conventional visual analysis

**Table:**

Parameter	Without reduced bowel enhancement (*n* = 10)	With reduced bowel enhancement (*n* = 22)	*p* value
Arterial phase			
CT values in 70-keV (HU)	48.2 ± 14.8	35.7 ± 12.9	0.022*
CT values in 40-keV (HU)	111.6 ± 42.4	78.2 ± 33.4	0.017*
Iodine quantity (μg/cm^3^)	11.6 ± 5.8	6.1 ± 4.0	0.001*
Venous phase			
CT values in 70-keV (HU)	49.2 ± 12.3	41.7 ± 13.2	0.077
CT values in 40-keV (HU)	115.1 ± 30.5	87.6 ± 36.9	0.040*
Iodine quantity (μg/cm^3^)	12.6 ± 4.6	7.8 ± 4.0	0.004*

*Statistically significant (*p* 0.05)
